# Dystrophic calcification and heterotopic ossification in fibrocartilaginous tissues of the spine in diffuse idiopathic skeletal hyperostosis (DISH)

**DOI:** 10.1038/s41413-020-0091-6

**Published:** 2020-04-02

**Authors:** Dale E. Fournier, Patti K. Kiser, Ryan J. Beach, S. Jeffrey Dixon, Cheryle A. Séguin

**Affiliations:** 1 Health and Rehabilitation Sciences (Physical Therapy), Faculty of Health Sciences, The University of Western Ontario, London, ON N6A 5B9 Canada; 2 Bone and Joint Institute, The University of Western Ontario, London, ON N6G 2V4 Canada; 3 Department of Laboratory Medicine and Pathology, Schulich School of Medicine & Dentistry, The University of Western Ontario, London, ON N6A 5C1 Canada; 4 Department of Physiology and Pharmacology, Schulich School of Medicine & Dentistry, The University of Western Ontario, London, ON N6A 5C1 Canada

**Keywords:** Bone, Pathogenesis

## Abstract

Diffuse idiopathic skeletal hyperostosis (DISH) is a prevalent noninflammatory spondyloarthropathy characterized by ectopic mineral formation along the anterolateral aspect of the vertebral column, yet little is known about its underlying pathogenesis. Our objective was to evaluate the histopathological features and composition of ectopic mineral within spinal tissues affected by DISH in humans. Thoracic spine segments from six embalmed cadaveric donors (one female and five males; median age 82 years) meeting the radiographic diagnostic criteria for DISH were evaluated using radiological, histological, and physical analyses. Overall, the histological features of ectopic mineralization at individual motion segments were heterogeneous, including regions of heterotopic ossification and dystrophic calcification. Heterotopic ossifications were characterized by woven and lamellar bone, multifocal areas of metaplastic cartilage, and bony bridges along the anterior aspect of the intervertebral disc space. Dystrophic calcifications were characterized by an amorphous appearance, a high content of calcium and phosphorus, an X-ray diffraction pattern matching that of hydroxyapatite, and radiodensities exceeding that of cortical bone. Dystrophic calcifications were found within the anterior longitudinal ligament and annulus fibrosus in motion segments both meeting and not meeting the radiographic criteria for DISH. In summary, our findings indicate that in DISH, ectopic mineral forms along the anterior aspect of the spine by both heterotopic ossification and dystrophic calcification of fibrocartilaginous tissues. Although both types of ectopic mineralization are captured by current radiographic criteria for DISH, dystrophic calcification may reflect a distinct disease process or an early stage in the pathogenesis of DISH.

## Introduction

Diffuse idiopathic skeletal hyperostosis (DISH) is a noninflammatory spondyloarthropathy characterized by bony outgrowths or hyperostoses along the anterolateral aspect of the vertebral column, particularly in the thoracic region.^1^ DISH is diagnosed by the radiographic detection of flowing mineral formation along four contiguous vertebral bodies, the preservation of intervertebral disc (IVD) height in the involved areas, and the absence of bony ankylosis of the vertebral facet and/or sacroiliac joints.^2^ Although not included in the diagnostic criteria, DISH is often associated with the presence of extraspinal hyperostoses, commonly in the knee, ankle, hip, shoulder, and elbow joints.^3^ Symptoms associated with DISH are variable, ranging from spine stiffness and decreased spinal ranges of motion (with or without back pain) to, in severe cases, dysphagia, spinal cord/nerve root compression, and vertebral fracture.^4–6^ Notably, the clinical symptoms are poorly understood, and the radiographic diagnosis of DISH is limited to an advanced disease state.^7^

The prevalence of DISH in North America and Europe is estimated to be 15%–25% and 17% of the population over the age of 50, respectively.^8,9^ Risk factors for DISH include ethnicity (e.g., Caucasians),^8^ sex (males > females),^8,9^ advanced age,^8,10^ and metabolic disorders (e.g., obesity, diabetes mellitus).^11^ Together, the lack of early detection of DISH, the rise in potential risk factors, and the unfamiliarity with DISH among medical professionals suggest that its prevalence is greater than previously reported.

The cause and biological pathways controlling the formation of ectopic mineral in DISH are unknown. Systemic metabolic changes related to obesity, diabetes mellitus, large waist circumference, hypertension, hyperinsulinemia, dyslipidemia, and hyperuricemia are associated with DISH.^4,11–14^ Familial cases of DISH,^15,16^ although rare, paired with the characterization of ectopic spine mineralization in animal models,^17–20^ suggest the contribution of genetic factors in the etiology of DISH. A related disorder, ossification of the posterior longitudinal ligament, has been reported frequently with DISH and is postulated to share a similar pathogenesis.^21^ DISH is characterized by the right-sidedness of the mineral formation in the thoracic spine, which is thought to be a consequence of the mechanical pressure created by persistent aortic pulsations on the left serving to inhibit soft tissue mineralization.^22^ Ultimately, our limited understanding of the pathobiology of DISH has resulted in the lack of early diagnostic indicators, prognostic factors, and disease-modifying treatments.^23^

The hallmark DISH characteristic of flowing ectopic spinal mineralization is based on radiographic description.^2^ The histopathological characteristics of DISH are less well known. Initial reports described histopathological features of endochondral ossification of the anterior longitudinal ligament^24^ as well as changes in the gross features of the IVD.^21,25^ In contrast, recent studies in cadaveric tissues demonstrated that the anterior longitudinal ligament was morphologically normal in appearance and was displaced by ectopic mineral, but did not itself undergo aberrant mineralization.^26^ Taken together, these findings underscore the need to identify and characterize the composition of the ectopic mineral formed in DISH, as well as the tissue types affected and associated cellular changes. The current study investigated these questions by combining radiological, histological, and physical analyses of human spine segments affected by DISH.

## Results

**Fig. 1:**
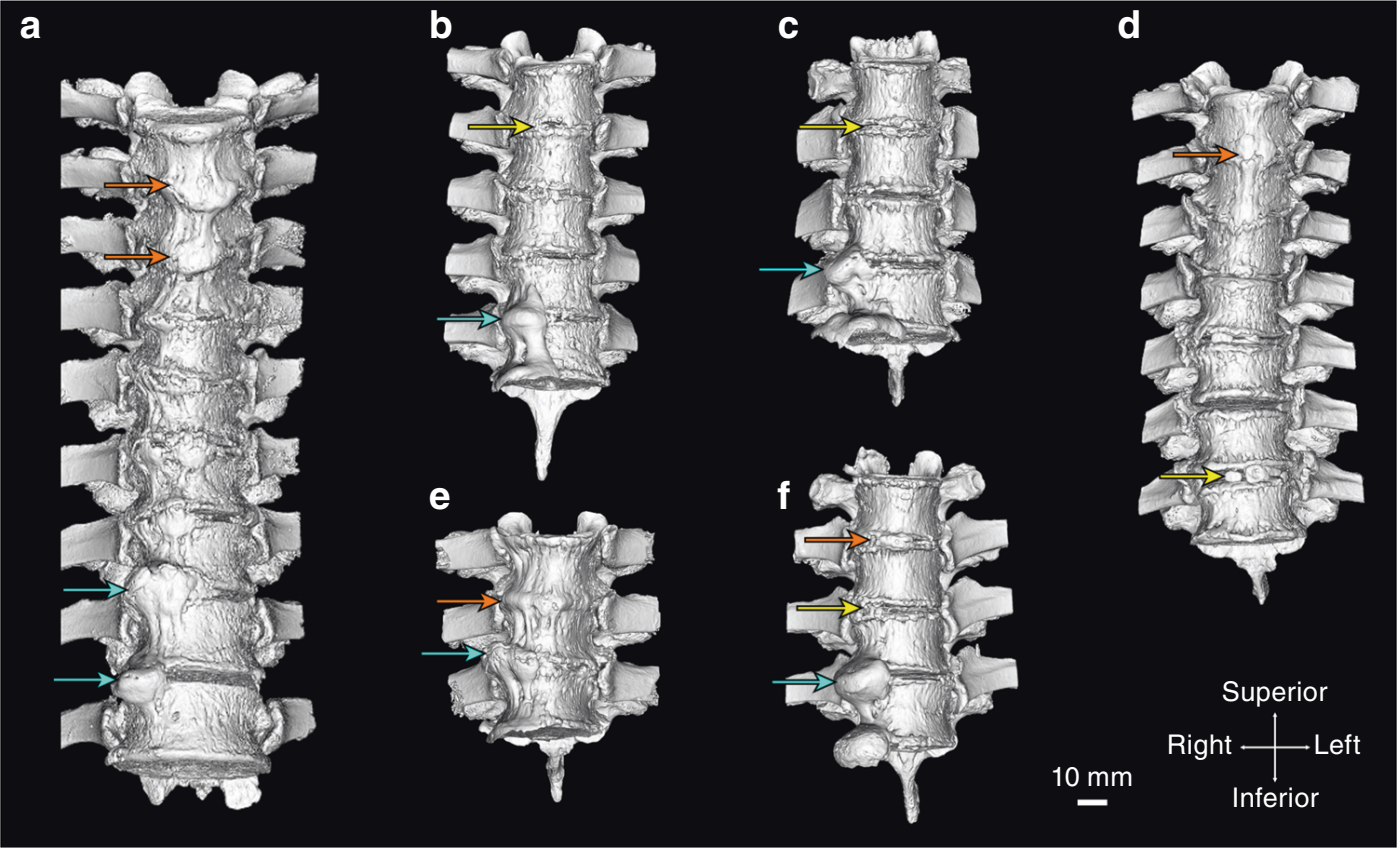
Three-dimensional isosurface renderings (anterior view) derived from microcomputed tomography (µCT) showing mineralized tissues (>310 Hounsfield units) of the spines included in the study. **a** 85-year-old male, thoracic vertebrae 4–11. **b** 77-year-old male, thoracic vertebrae 3–7. **c** 88-year-old male, thoracic vertebrae 7–9. **d** 72-year-old male, thoracic vertebrae 3–9. **e** 78-year-old male, thoracic vertebrae 4–6. **f** 86-year-old female, thoracic vertebrae 4–7. Arrows indicate the level of the intervertebral disc of the specific motion segments included in the study; yellow arrows—discontinous-patchy presentation, orange arrows—vertical presentation, and cyan arrows—horizontal presentation. The scale bar represents 10 mm

A previous study by our group characterized the morphometry and radiodensity of ectopic mineral associated with DISH in embalmed cadaveric human spines using microcomputed tomography (µCT) imaging.^27^ Mineral formations at individual motion segments in the thoracic spine were differentiated based on morphology, categorized as vertical bands when the resultant bridging angle relative to the vertebrae was greater than 90°, as horizontal outgrowths when the resultant bridging angle was <90°, or as discontinuous-patchy when the mineral formed an incomplete bridge.^27^ For the present study, we carried out detailed analyses of 15 individual motion segments from the thoracic spine of six donors with DISH from the previous study (one female and five males; median age 82 years, range 72–87) (Fig. 1).^27^ These spines were selected based on (i) having met the diagnostic criteria for DISH (with at least four contiguous segments affected,^2^ previously assessed by two clinician observers) and (ii) each spine containing at least two of the three morphological presentations of ectopic mineralization described in our previous study (discontinuous-patchy, vertical, or horizontal).^27^

### Histological features of spinal tissues

**Fig. 2:**
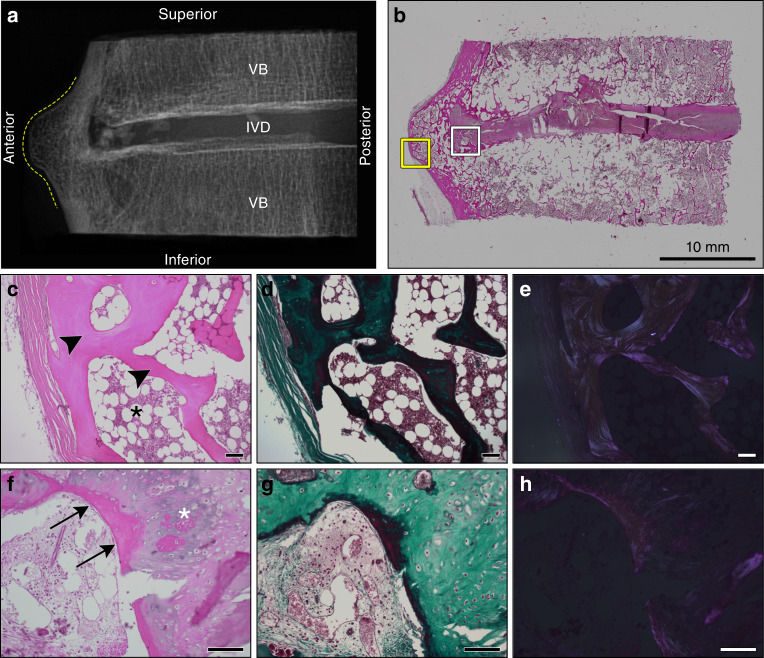
Histological appearance of a representative motion segment with horizontal presentation of ectopic mineralization associated with DISH. Images correspond to the specimen shown in Fig. 1b, T6-7. **a** Digital radiograph of the intact tissue prior to decalcification showing the localization of ectopic mineral within the motion segment (contoured by dotted yellow line). VB vertebral bone, IVD intervertebral disc. **b** Representative section stained with hematoxylin and eosin demonstrating the appearance of the intact section; scale bar represents 10 mm. The yellow box corresponds to an anterior region with features of heterotopic ossification imaged with a 10× objective (**c**–**e**), while the white box corresponds to a transition region from heterotopic ossification to the underlying fibrocartilage annulus fibrosus imaged with a 20× objective (**f**–**h**). **c**, **f** stained with hematoxylin and eosin; **d**, **g** stained with Masson’s trichrome; **e**, **h** the hematoxylin and eosin stained sections shown in **c** and **f** visualized with polarized light. Scale bars for **c**–**h** represent 100 µm. **c**–**e** Highlight features of heterotopic ossification along the anterior aspect of the motion segment, including compartmentalized bone marrow cavity (black asterisk) and organized lamellar bone (black arrowheads). **f**–**h** Characteristics of primary woven bone (black arrows) and organized nests of chondrocytes (white asterisk) associated with endochondral ossification. All images are oriented as shown in **a**

**Fig. 3:**
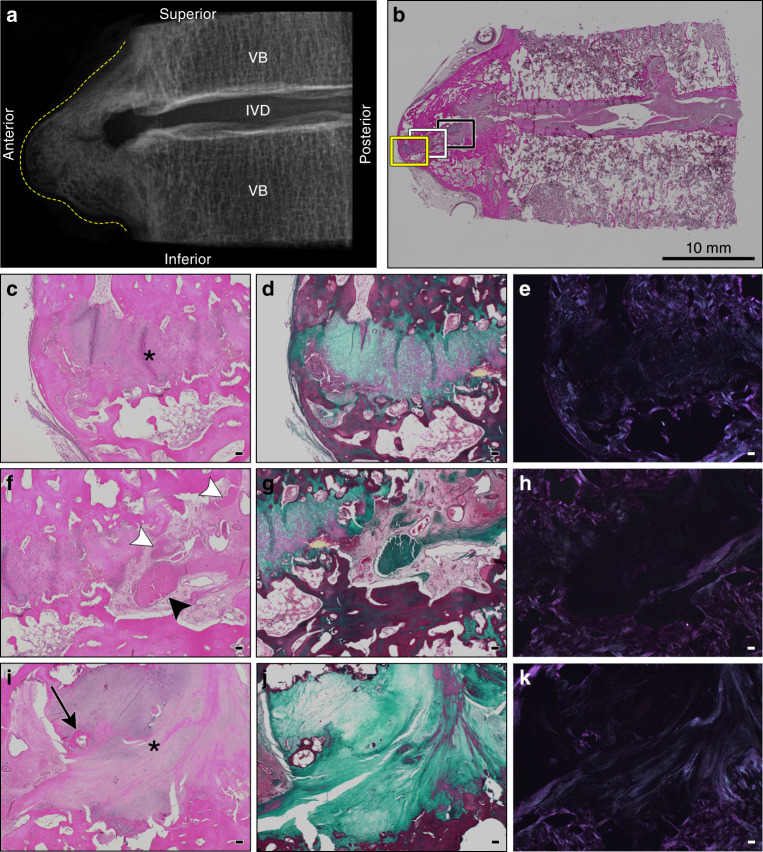
Histological appearance of a representative motion segment with horizontal and discontinuous-patchy presentations of ectopic mineralization associated with DISH. Images correspond to the specimen shown in Fig. 1c, T8-9. **a** Digital radiograph of the intact tissue prior to decalcification showing the localization of ectopic mineral within the motion segment (contoured by dotted yellow line). VB vertebral bone, IVD intervertebral disc. **b** Representative section stained with hematoxylin and eosin demonstrating the appearance of the intact section; scale bar represents 10 mm. Yellow (**c**–**e**), white (**f**–**h**), and black boxes (**i**–**k**) correspond to regions of fibrocartilage extending from the IVD positioned between mineralized outgrowths imaged with a 4× objective. **c**, **f**, **i** stained with hematoxylin and eosin; **d**, **g**, **j** stained with Masson’s trichrome; and **e**, **h**, and **k** the hematoxylin and eosin stained sections visualized with polarized light. Scale bars for **c**–**k** represent 100 µm. **c**–**e** Highlight the anterior-most portion of the fibrocartilaginous extension (asterisk). **f**–**h** Reveal a unique transition zone marked by the presence of amorphous granular material (black arrowhead) and multifocal areas of fibrosis (white arrowheads). **i**–**k** Display the fibrocartilage region (asterisk) adjacent to the native annulus fibrosus and a localized region of ossification (black arrow). All images are oriented as shown in **a**

**Fig. 4:**
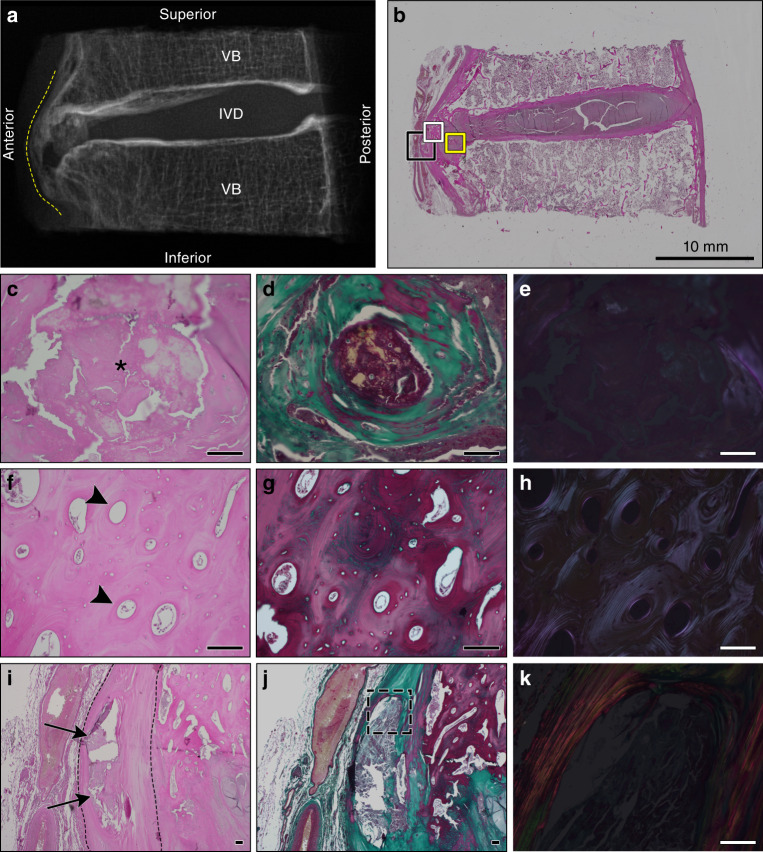
Histological appearance of a representative motion segment with vertical presentation of ectopic mineralization associated with DISH. Images correspond to the specimen shown in Fig. 1a, T4–5. **a** Digital radiograph of the intact tissue prior to decalcification showing the localization of ectopic mineral within the motion segment (contoured by dotted yellow line). VB vertebral bone, IVD intervertebral disc. **b** Representative section stained with hematoxylin and eosin demonstrating the appearance of the intact section; scale bar represents 10 mm. The yellow box corresponds to the region within the fibrocartilage of the IVD with a discrete amorphous calcification imaged with a 20× objective (**c**–**e**). The white box corresponds to a region of heterotopic ossification along the anterior aspect of the IVD imaged with a 20× objective (**f**–**h**). The black box corresponds to the discrete calcification within the anterior longitudinal ligament imaged with 4× and 20× objectives (**i**–**k**). **c**, **f**, **i** Stained with hematoxylin and eosin; **d**, **g**, **j** stained with Masson’s trichrome; and **e**, **h**, **k** the hematoxylin and eosin stained sections visualized with polarized light. Scale bars for **c**–**k** represent 100 µm. **c**–**e** Highlight an amorphous region of dystrophic calcification localized within the annulus fibrosus (asterisk) and characterized by a granular appearance and variable eosinophilic staining. **f**–**h** A region of heterotopic ossification marked by organized osteons (black arrowheads). **i**–**k** Reveal an area of dystrophic calcification (black arrows) localized within the anterior longitudinal ligament (contoured by black dotted lines). **k** Corresponds to the dotted outline in **j**. All images are oriented as shown in **a**

Twelve regions of the spine containing ectopic mineral structures at individual motion segments were isolated (Supplementary Fig. S1) to assess the histological appearance of affected tissues. Figure 2 is representative of a motion segment with the morphological features of “horizontal” and “discontinuous-patchy” mineralization; Fig. 3 is representative of a motion segment with the morphological features of “horizontal” mineralization; and Fig. 4 is representative of a motion segment with the morphological features of “vertical” mineralization. As a reference, Supplementary Fig. S2 is representative of a motion segment with no evidence of ectopic mineralizations from a spine that did not meet the diagnostic criteria for DISH. In Figs. 2–4, digital radiographs are paired with low-magnification views of the intact motion segment to correlate histological features with areas of tissue mineralization within decalcified tissue sections. Specific areas of interest are presented at higher magnification, with serial sections stained with hematoxylin and eosin to localize cells and evaluate extracellular matrix structure, Masson’s trichrome to detect the collagenous component of the extracellular matrix, and later visualized by polarized light microscopy to assess birefringence as an indicator of optical isotropy.

IVDs within the intact motion segments examined demonstrated consistent histological features (*n* = 12: Figs. 2–4). Although radiographic IVD height was maintained in keeping with Resnick’s criteria,^2^ the nucleus pulposus was granular in appearance, indicative of mild-to-moderate degeneration. In all sections examined, varying degrees of IVD degeneration were observed. The characteristics of degeneration included the loss of a distinct transition between the nucleus pulposus and surrounding annulus fibrosus (Figs. 2b and 4b) as well as moderate-to-severe disorganization of the lamellar structure of the annulus fibrosus, most often detected along the anterior aspect of the IVD (Fig. 3b). The cartilage endplates showed irregularities, ranging from the loss of organization of the cartilaginous matrix to small clefts and the disruption of the subchondral bone (i.e., Schmorl’s nodes, detected in 3/12 motion segments from 3/6 specimens; Figs. 2b and 3b). The degenerative changes detected in the IVD were expected given the advanced age of donors (range 72–87 years). The anterior longitudinal ligament (when in-section) was either well preserved or contained discrete regions of mineralization (Fig. 4).

### Histological features of ectopic mineral structures

In all motion segments assessed, histological examination confirmed the radiographic selection criterion of a mineralized tissue bridge between the superior and inferior vertebrae across an IVD (Figs. 2–4). In general, cellular indications of inflammation were absent from areas of ectopic mineralization, although small areas of scattered inflammatory cells (primarily macrophages, lymphocytes, and plasma cells) and neovascularization were noted. Tremendous heterogeneity was, however, noted in the histological features of the ectopic mineral structures associated with DISH.

First, the ectopic mineral bridges between motion segments associated with the radiographic “flowing candle wax” appearance of DISH varied in length, thickness, volume, and the type of bone (woven vs. mature). In motion segments characterized by µCT as either vertical or horizontal morphological presentations, the ectopic bridges showed features of well-developed lamellar bone, consistent with heterotopic ossification (Figs. 2c–e and 4f–h). The presence of organized osteons was confirmed by polarized light microscopy (Figs. 2e and 4h). In some motion segments, these areas of heterotopic bone also contained bone marrow spaces (Fig. 2c–e). Often noted were focal areas of fibrosis and primary woven bone adjacent to or within areas of mature heterotopic bone (Fig. 2f–h).

Second, although motion segments with ectopic mineral presenting as horizontal outgrowths showed similar radiographic appearances (Figs. 1, 2a, and 3a), the large ectopic bone mass was not always continuous. Instead, areas of fibrocartilage were often detected in this region, extending from the IVD and positioned between the superior and inferior mineralized outgrowths (Fig. 3b–k). Within these fibrocartilage regions were multifocal areas of granular degeneration, fibrosis, and ossification. These sites showed a transition from areas of fibrocartilage to nests of chondrocytes within cartilage to sites of woven bone, consistent with the process of endochondral ossification.

Last, isolated regions of amorphous calcified material were consistently identified in motion segments corresponding to all the morphological presentations of ectopic mineralization associated with DISH (i.e., discontinuous-patchy, vertical, and horizontal). These regions showed features consistent with dystrophic calcification, which occurs in damaged soft tissues and is characterized by amorphous deposits of calcium phosphate.^28,29^ Areas of dystrophic calcification were detected by histology and were localized within (i) the fibrocartilaginous tissues located between the IVD and areas of heterotopic ossification; (ii) the annulus fibrosus (Fig. 4c–e); or (iii) the anterior longitudinal ligament (Fig. 4i–k). These areas of dystrophic calcification were often variably stained and granular in appearance, which may indicate a mixture of calcified material and degenerated fibrocartilage or differences in the organic constituents of the mineralized matrix. The origin of this material could not be determined. Polarized light microscopy of regions of dystrophic calcification showed little birefringence, consistent with a lack of structural organization (Fig. 4e, k).

### Elemental composition and X-ray diffraction patterns of ectopic mineral

**Fig. 5:**
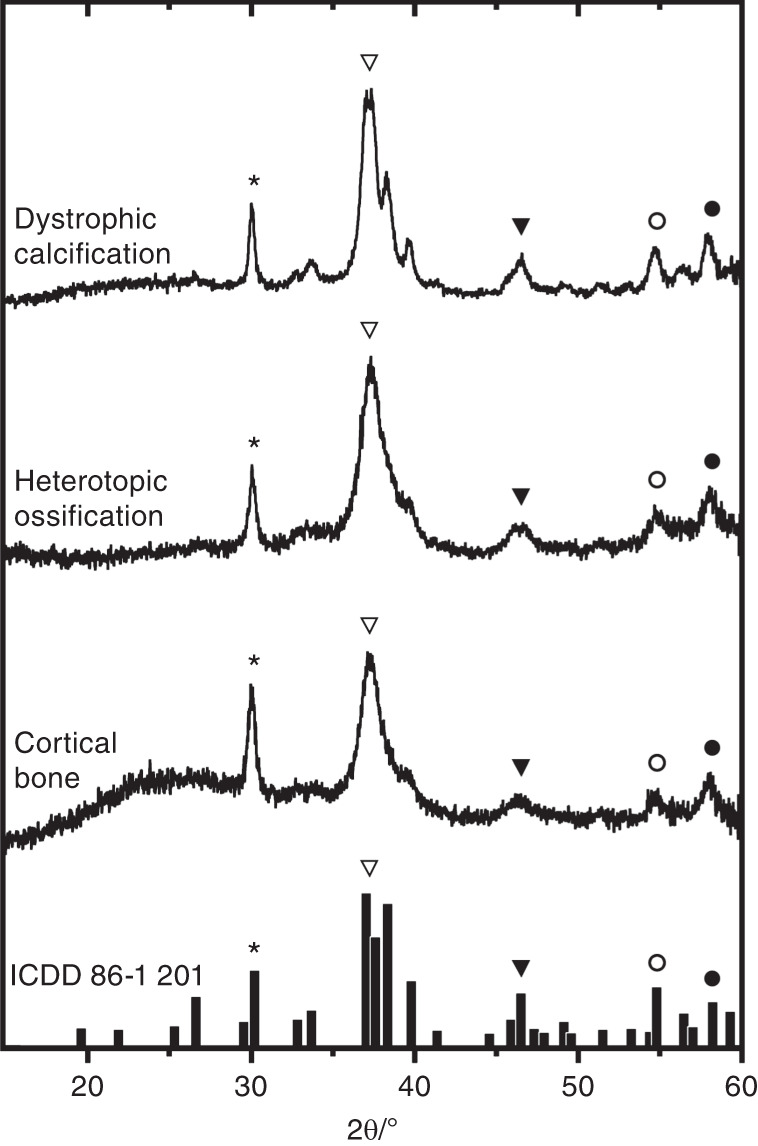
X-ray diffraction patterns from regions of ectopic mineralization associated with DISH. Shown are representative X-ray diffraction patterns from individual regions within sites of dystrophic calcification (*n* = 2 spines; 2 regions per deposit), heterotopic ossification (*n* = 3 spines; 2–4 regions per deposit), and unaffected cortical bone (3 spines; 1 region per segment) displayed in a stack plot. The results were matched to calcium-deficient hydroxyapatite [Ca_8.8_(PO_4_)_6_(OH)_1.92_] from the International Centre for Diffraction Data (powder diffraction file: 86–1 201), as indicated by the bar plot at the bottom and symbols

Given that areas of ectopic mineralization associated with DISH contained histological features consistent with both heterotopic ossification and dystrophic calcification, we sought to determine and compare the mineral composition of these structures. First, regions of interest within four individual motion segments from two spines meeting the diagnostic criteria for DISH (Fig. 1a, f) were analyzed by energy-dispersive X-ray spectroscopy to determine the elemental composition in areas of heterotopic ossification (*n* = 5–10 regions/motion segment) and dystrophic calcification (*n* = 3–8 regions/motion segment). The calcium content was found to be significantly greater in all sites of ectopic mineralization associated with DISH (heterotopic ossification and dystrophic calcification) than in unaffected vertebral bone (indicated as cortical bone) (Table 1). Sites of dystrophic calcification were associated with a greater phosphorus content compared to unaffected vertebral bone and showed greater calcium and phosphorus content compared with regions of heterotopic ossification. The calcium/phosphorus ratio was significantly greater in all regions of ectopic mineralization than in unaffected vertebral bone. On the other hand, the calcium/phosphorus ratios were similar at sites of heterotopic ossification and dystrophic calcification. Next, to assess the crystalline structure within these regions, tissues were analyzed by X-ray diffraction. Regions of interest within sites of dystrophic calcification and heterotopic ossification showed an X-ray diffraction pattern matching that of calcium-deficient hydroxyapatite [Ca_8.8_(PO_4_)_6_(OH)_1.92_, powder diffraction file: 86-1201, International Centre for Diffraction Data, 2018], as did regions within unaffected cortical bone (Fig. 5). The theoretical calcium/phosphorus ratio for calcium-deficient hydroxyapatite is 1.47, close to the values found by energy-dispersive X-ray spectroscopy (Table 1).

**Table 1:** Calcium and phosphorus content as determined by energy-dispersive X-ray spectroscopy

Tissue	*n*	Percent atomic ratio		Ca/P ratio
		Calcium	Phosphorus	
Dystrophic calcification	18	8.85^a,b^ (1.30)	5.65^a,b^ (0.90)	1.57^a^ (0.05)
Heterotopic ossification	34	7.03^a^ (0.89)	4.53 (0.53)	1.55^a^ (0.05)
Cortical bone	13	6.05 (0.48)	4.13 (0.26)	1.47 (0.06)

Calcium and phosphorus content were determined by energy-dispersive X-ray spectroscopy and Ca/P ratio was calculated. Data are means (standard deviations) for regions of dystrophic calcification (2 spines; 18 regions total), heterotopic ossification (3 spines; 34 regions total), and unaffected cortical bone (3 spines; 13 regions total).

^a^Significantly greater than corresponding parameter for cortical bone (*P* < 0.05).

^b^Significantly greater than corresponding parameter for heterotopic ossification (*P* < 0.05); as determined using one-way ANOVA with Bonferroni’s multiple comparisons test.

### Structure and radiodensity analysis of ectopic mineral

**Fig. 6:**
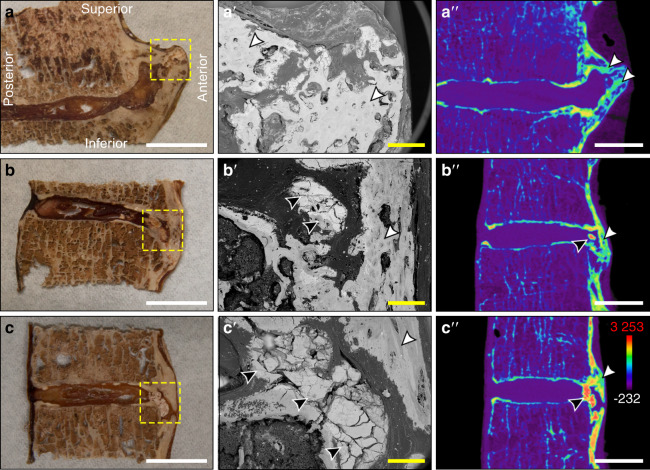
Characterization of the gross morphology and radiodensities of ectopic mineralizations associated with DISH. **a**–**aʹʹ** Thoracic segment T9-10 from an 85-year-old male with heterotopic ossification (Fig. 1a), **b**–**bʹʹ** thoracic segment T3-4 from a 72-year-old male with dystrophic calcification (Fig. 1f), and **c**–**cʹʹ** thoracic segment T3-4 from an 85-year-old male with dystrophic calcification (Fig. 1a). **a**–**c** The gross appearance of desiccated tissues with yellow dotted lines showing regions analyzed by scanning electron microscopy; white scale bars represent 10 mm. **aʹ**–**cʹ** Corresponding scanning electron microscopy images; yellow scale bars represent 1 mm. **aʹʹ**–**cʹʹ** Pseudocolored radiodensity maps from matched µCT slices. Colors represent various radiodensities: purple (−232 to 310 Hounsfield units) corresponds to soft tissues; blue–green (310–2 750 Hounsfield units) corresponds to normal bone; and orange–red (2 750–3 253 Hounsfield units) corresponds to mineralizations exceeding the density of normal cortical bone. Topographical analysis of tissue sections by scanning electron microscopy highlighted the uniform, amorphous appearance of dystrophic calcifications (black arrowheads) within the intervertebral disc, which are distinct in appearance from adjacent ossifications (white arrowhead); white scale bars represent 10 mm. All images are oriented as shown in **a**

Ectopic mineral structures associated with DISH in individual motion segments were further characterized by scanning electron microscopy and µCT (Fig. 6). An examination of areas of dystrophic calcification by scanning electron microscopy revealed distinct regions with a uniform, amorphous structure that consistently appeared fractured following tissue processing, distinct from the appearance of adjacent cortical bone (Fig. 6b, c). Similar to findings from the histopathological analyses, these regions were localized to the annulus fibrosus of the IVD as well as the fibrocartilaginous structures adjacent to heterotopic ossifications. Finally, µCT was used to generate pseudocolored radiodensity maps of the same regions assessed for physical characteristics. This analysis demonstrated remarkable variation in the radiodensity of mineralized structures (Fig. 6). Within motion segments examined, foci of mineralized tissues corresponding to regions of dystrophic calcification were detected, with radiodensities that exceeded those corresponding to regions of heterotopic ossification and unaffected vertebral bone. The presence of these regions of high radiodensity is consistent with the greater calcium and phosphorus content found in dystrophic calcifications using energy-dispersive X-ray spectroscopy.

Having identified hyperdense regions (with radiodensities exceeding those of normal cortical bone) as a feature of dystrophic calcification, we used this as a criterion to evaluate the presence of dystrophic calcification in spines not diagnosed with DISH and their association with mineralization bridging the IVD. Using µCT, we first evaluated the presence or absence of hyperdense mineralized foci within the annulus fibrosus or anterior longitudinal ligament in 77 motion segments from spines that did not meet the diagnostic criteria for DISH and 94 motion segments from spines with DISH (Table 2). One spine with advanced bridging associated with ankylosing spondylitis was excluded from the analysis to reduce false positives. We showed that in spines that do not meet the diagnostic criteria for DISH, 38 of 77 motion segments (49%) had hyperdense foci within the IVD, compared with 72 of 94 motion segments (77%) in spines with DISH. Although dystrophic calcifications are not a unique feature associated with DISH, they are more prevalent in motion segments from spines diagnosed with DISH than in those from spines not diagnosed with DISH (*P* = 0.000 4). We also examined whether the presence of these hyperdense foci was associated with the presence of mineralization bridging the IVD (heterotopic ossification). In spines that did not meet the diagnostic criteria for DISH, 12 of 38 motion segments with hyperdense foci (32%) also had mineralization bridging the IVD; in contrast, in spines with DISH, 58 of 72 motion segments with hyperdense foci (80%) also had mineralization bridging the IVD. Statistical analysis demonstrated that the presence of hyperdense foci was associated with mineralization bridging the IVD in both groups.

**Table 2:** Prevalence of hyperdense mineralized foci in all motion segments with or without ectopic mineral bridging of the IVD as determined by µCT, in spines that meet or do not meet the diagnostic criteria for DISH

IVD	Spines meeting the criteria for DISH (*n* = 9)			Spines *not* meeting the criteria for DISH (*n* = 9)		
	Hyperdense foci			Hyperdense foci		
	Yes	No	Total	Yes	No	Total
Bridge	58	9	67	12	4	16
No Bridge	14	13	27	26	35	61
Total	72	22	94	38	39	77
	Two-sided, Fisher’s exact test (α = 0.05); *P* = 0.000 8			Two-sided, Fisher’s exact test (α = 0.05); *P* = 0.026 3		

Data are binary counts (yes/no) for the presence of hyperdense foci within individual motion segments related to the presence or absence of ectopic mineral bridging the IVD. Data are presented for two categories, spines that meet or do not meet the diagnostic criteria for DISH

This analysis is importantly limited by the diagnostic criteria for DISH used to categorize the specimens. Specifically, the diagnosis of DISH requires four continuous motion segments with radiographic evidence of bridging mineralization, a threshold thought to be associated with an advanced stage of disease. In fact, some of the spines that did not meet the diagnostic criteria for DISH showed ectopic mineralization bridging more than one but less than four motion segments. To eliminate potential variability associated with the stage of DISH, we analyzed the association between the presence of hyperdense foci and mineralization bridging the IVD in all motion segments, independent of the classification as DISH (Table 3). Of the 171 motion segments assessed, 110 showed hyperdense foci within the IVD. Of those, 70 (64%) had mineralization bridging the IVD, demonstrating a strong association between these two features (*P* < 0.000 1).

**Table 3:** Prevalence of hyperdense mineralized foci in all motion segments with and without ectopic mineral bridging of the IVD as determined by µCT

IVD	All available motion segments (*n* = 18 spines)		
	Hyperdense foci		
	Yes	No	Total
Bridge	70	13	83
No bridge	40	48	88
Total	110	61	171

Two-sided, Fisher’s exact test (*α* = 0.05); *P* < 0.000 1

Data are binary counts (yes/no) for the presence of hyperdense foci within individual motion segments related to the presence or absence of ectopic mineral bridging of the IVD, irrespective of categorization based on the diagnostic criteria for DISH

## Discussion

The features of ectopic mineral formation that define DISH have been primarily characterized through clinical imaging, with few previous reports containing detailed characterization of the tissue-specific changes within spinal motion segments. The present investigation addressed this gap by characterizing the pathological features of ectopic mineral structures associated with DISH in human cadaveric tissues using a combination of radiological, histological, and physical approaches. Our findings are the first to establish that ectopic mineralization in DISH results from both heterotopic ossification and dystrophic calcification of spinal tissues, including the IVD and anterior longitudinal ligament. Based on these and previous studies from our group,^27^ we postulate that the two forms of ectopic mineralization may reflect different disease processes or perhaps distinct stages in the pathogenesis of DISH.

The classic radiographic feature of DISH is the presence of flowing mineralization along the anterolateral aspect of the vertebral column,^1^ but our previous report identified heterogeneity in the morphological appearance of ectopic mineral associated with DISH based on µCT analysis.^27^ This heterogeneity was likewise evident in the histological appearance of individual motion segments from spines with DISH based on variability noted in the structure, organization, and features of the mineralized bridges along the anterior aspect of the spine. For example, despite demonstrating a similar radiographic appearance, variability was detected in the features of mature lamellar bone (with or without bone marrow) and indicators of endochondral ossification. Importantly, our study also identified discrete regions of amorphous, acellular calcified material within the outer lamellae of the annulus fibrosus, the anterior longitudinal ligament, and fibrocartilage extending from the IVD adjacent to anterolateral bony bridges. These regions showed histological features consistent with dystrophic calcification and were detected regardless of the morphological classification of the ectopic mineral structure (i.e., discontinuous-patchy, vertical, and horizontal presentations). Regions of dystrophic calcification were histologically distinct from areas of heterotopic ossification and demonstrated differences in calcium and phosphorus content; on the other hand, both showed X-ray diffraction patterns matching that of calcium-deficient hydroxyapatite. Consequently, DISH may not solely be a pathology of bone formation (heterotopic ossification) but also may include features of dystrophic calcification. Notably, µCT analysis of areas of dystrophic calcification demonstrated radiodensities exceeding those in unaffected vertebral bone and in areas of heterotopic ossification. These hyperdense foci of dystrophic calcifications were present in all three of the described morphological presentations of disease and were associated with the presence of ectopic mineralization bridging the IVD. While it is tempting to speculate that these distinct histopathological features of dystrophic calcification may reflect different stages of disease, further studies are required to assess their relation to the spatiotemporal pattern of disease progression.

Important to the diagnosis of DISH is the exclusion of other spine pathologies, such as spondylosis (i.e., IVD degeneration) and ankylosing spondylitis.^2^ Specifically, in contrast to spondylosis, IVD height is preserved, and osteophytes are vertical and bridging in DISH, whereas in spondylosis, they are usually transverse, based on radiographic evaluation.^30^ A pilot study conducted with spinal tissues from ten cadavers with DISH established that the degree of histopathological IVD degeneration and measures of disc height were comparable between age- and sex-matched specimens without DISH, suggesting a limited role for IVD degeneration in the pathogenesis of DISH.^25^ The histopathological evaluation in the current study demonstrated mild-to-moderate IVD degeneration in all specimens examined, which may have been insufficient to affect radiographic disc height. Note that loss of IVD height would preclude the diagnosis of DISH based on radiographic criteria.^2^ These findings are in keeping with previous studies demonstrating degenerative changes in the IVDs of people with DISH.^31,32^ Taken together, mild-to-moderate IVD degeneration should be reconsidered as an accompanying clinical scenario in the formation of ectopic mineral in DISH, since it is common in this population.

Despite the radiographic preservation of IVD height (with or without mild-to-moderate degeneration), the current study demonstrated that morphological changes to the outer annulus fibrosus are frequent in DISH. Our findings agree with those of previous studies that identified fibrocartilage extending from the IVD between the anterolateral bony bridges associated with both the superior and inferior vertebrae, lying adjacent to the anterior longitudinal ligament.^25,33^ In fact, Kuperus et al. proposed a scoring system to characterize the gross morphological shape of these fibrocartilaginous extensions from the IVD (defined as regular, tapered, spatulate, or irregular^29^—several of which were noted in the current investigation). In our study, histopathological evaluation of these fibrocartilaginous regions revealed features of endochondral ossification, including nests of chondrocytes and primary woven bone, as well as distinct areas of dystrophic calcification.

We theorize that dystrophic calcification may be the result of cellular changes or necrosis of resident fibrocartilage cells in response to trauma, degeneration, or age. In this regard, injury to soft tissues, such as cardiovascular and connective tissues, including tendons, can lead to local aberrant production or unmasking of molecules that serve as substrates to nucleate the initial formation of calcium phosphate crystals within the extracellular matrix, leading to dystrophic calcification.^34,35^ It is possible that dystrophic calcifications within the soft tissue structures of the spine (i.e., annulus fibrosus and anterior longitudinal ligament) play a role in the pathogenesis of DISH. There are several potential mechanisms by which dystrophic calcification could lead to heterotopic ossification. For example, it is known that calcium phosphate itself within soft tissue can induce ossification,^36–38^ given the presence of appropriate precursor cells and microenvironmental conditions. Thus, dystrophic calcification could directly lead to heterotopic ossification in spinal tissues. It is possible that within the spinal ligaments and periphery of the IVD, the right conditions exist to permit ossification. In contrast, within the deeper lamellae of the annulus fibrosus, dystrophic calcifications might persist without transitioning to heterotopic ossification due to the lack of appropriate osteogenic precursors or an osteoconductive microenvironment. A second potential mechanism is that dystrophic calcification in spinal fibrocartilage leads to altered tissue biomechanics. Increased tissue stiffness, due to the presence of dystrophic calcification, may induce the formation of osteophytes. In this regard, others have proposed that increased mechanical stress on vertebrae can lead to the formation of osteophytes.^39^ It is conceivable that these osteophytes may then lead to bridging ossification of the spinal ligaments and the periphery of the IVD. This possibility is in keeping with previous studies proposing that DISH is associated with degenerative osteophyte formation, similar to that occurring in spondyloses and osteoarthritis.^4^ To resolve the mechanism, we require further studies of the soft tissues of the spine at early stages of ectopic mineralization.

There are mixed reports regarding the involvement of the anterior longitudinal ligament in the pathogenesis of DISH. Some studies have reported mineralization of the anterior longitudinal ligament in DISH,^21,24,40^ whereas others report that the anterior longitudinal ligament does not undergo mineralization^1,21^ or that it is displaced by the formation of ectopic mineral structures.^26^ In the current study, when present in the histological sections examined, the structure of the anterior longitudinal ligament was typically preserved. However, in a subset of specimens, we detected focal regions of dystrophic calcification within the ligament, resembling those detected within the annulus fibrosus. Further studies are required to specifically examine the spatiotemporal association between the calcification of the IVD and the calcification of the anterior longitudinal ligament in the context of DISH progression and severity.

The findings from the current study establish that current radiographic criteria for DISH capture heterogeneous features associated with both dystrophic calcification and heterotopic ossification of fibrocartilaginous tissues of the spine. Taken together, the current investigation infers that DISH pathogenesis involves two distinct types of ectopic mineralization based on (i) the morphological appearance of the mineral; (ii) their calcium and phosphorus content; (iii) their radiodensity; and (iv) the tissues affected. Similar to calcific tendonitis, the detection of focal areas of dystrophic calcification within fibrocartilaginous tissues of the spine may serve as a radiographic indication of early-stage DISH. Future work is required to validate the association of these radiographic and histological features in a larger cohort of specimens, to correlate histological features with disease symptoms and/or comorbidities, to identify biomarkers to differentiate these presentations in clinical studies, and to associate clinical symptoms and disease features with the progression of the disease.

## Materials and methods

This study was conducted with intact embalmed human cadaveric spines (*n* = 19: 6 females and 13 males, median age 85 years, range 65–94) from the Schulich School of Medicine & Dentistry at The University of Western Ontario in accordance with the Anatomy Act of Ontario and guidelines of Western’s Committee for Cadaveric Use in Research (REB#10292018). The embalming process consisted of arterial distribution of embalming fluid, containing a mixture of ethanol, phenol, and formalin (Wessels & Associates: Troy, MI, USA), 24 to 48 h postmortem. The removal of the head and neck musculature exposed the first cervical vertebrae, and complete resection was performed inferior to the twelfth thoracic vertebral body. The ribs were dissected 3–5 cm lateral to the costovertebral joints, and soft tissues associated with the spine were preserved except for the descending thoracic aorta, which was removed.

### Microcomputed tomography (µCT) imaging

Intact human vertebral columns were previously scanned by µCT as described^27^ using a cone-beam X-ray imaging system (GE Locus eXplore Ultra: London, CAN) at a peak voltage of 80 kVp and tube current of 50 mA. The 1 000 X-ray projections were reconstructed into a single three-dimensional volume with an isotropic voxel spacing of 154 µm. Image volumes were rescaled into Hounsfield units using an internal calibrator of air and water and cortical bone substitute (450-SB3, Gammex RMI: Middleton, WI, USA). The µCT data were used to generate a series of images for each specimen that were previously assessed by two clinician observers to diagnose DISH using Resnick and Niwayama’s radiographic criteria.^2^ Three-dimensional isosurface renderings and pseudocolored µCT images were exported from MicroView (Version 2.2, GE Healthcare: London, CAN; and Version 2.5.0, Parallax Innovations Inc.: Ilderton, CAN). Data from µCT were grouped into contingency tables and assessed by two-sided Fisher’s exact test (*α* = 0.05).

### Histological evaluation

Six thoracic spines meeting the diagnostic criteria for DISH (one female and five males; median age 82 years, range 72–87) were dissected into individual motion segments by transverse cuts across the waist of the superior and inferior vertebrae to maintain the IVD and by oblique cuts to remove the posterior osseous features from the vertebral body (Supplemental Fig. S1a). Multiple motion segments from each specimen were selected for analyses based on the presence of distinct presentations of ectopic mineralization previously described by our group (i.e., discontinuous-patchy, vertical, and horizontal).^27^ A total of 15 motion segments were analyzed.

To isolate tissues of interest from the intact motion segments, a sagittal slice was made through the center of the ectopic mineralization associated with each motion segment using a rotating diamond blade saw (Supplemental Fig. S1b). In cases of right-sidedness, oblique cuts were made through the center of the ectopic mineralization (Supplemental Fig. S1c). From one of these halves, a 1-mm-thick slice of tissue was dissected for subsequent analysis of tissue composition. Each half of the isolated motion segments and the 1-mm-thick slices were imaged using a digital X-ray system (Planmeca ProX™: Helsinki, FIN) to localize areas of ectopic mineral. Based on the digital radiographs, one half was selected for histological analysis and decalcified with Shandon™ TBD-2™ (catalog no. 6764004, Thermo Scientific™: Nepean, CAN) for a duration of 14–21 days. Following decalcification, the tissues were processed and sectioned at 5 µm thickness using a Leica RM2255 microtome (Leica Biosystems Nußloch GmbH: Nußloch, DEU) and collected on 50 × 75 mm slides (Brain Research Laboratories: Newton, MA, USA). Serial sections were stained with hematoxylin and eosin to localize cells and to evaluate extracellular matrix structure, and Masson’s trichrome to detect the collagenous component of the extracellular matrix. Low-magnification micrographs were captured using a Cytation™ 5 Cell Imaging Multi-Mode Reader (BioTek Instruments, Inc.: Winooski, VT, USA), and high-magnification micrographs (including those under linear polarized light) were captured using an Olympus BX41 optical microscope equipped with a digital camera (Olympus U-TVO.5XC-3, Olympus Canada Inc.: Toronto, CAN) and Infinity Analyze software (Version 6.5.5, Lumenera Co.: Ottawa, CAN). Images were imported into Adobe® Photoshop® CC 2018 (Version 19.1.7, Adobe Systems Inc.: San Jose, CA, USA) for figure construction.

### Scanning electron microscopy, energy-dispersive X-ray spectroscopy, and X-ray diffraction

The 1-mm-thick slices of tissue taken from the center of the ectopic mineralization were desiccated with Drierite (W.A. Hammond Drierite Co.: Xenia, OH, USA) for 30 days, photographed with a Canon EOS 7D digital single-lens reflex camera, rinsed in 100% chloroform for 1 h, and subsequently redried for 1–2 days before coating with a 10 nm layer of osmium.^41^ Scanning electron microscopy was performed using a Zeiss 1540XB FIB/SEM instrument (Carl Zeiss: Oberkochen, DEU) at The University of Western Ontario Nanofabrication Facility. Energy-dispersive X-ray spectroscopy data were collected using an Oxford Instruments X-max50 analysis system and ICNA software (Oxford Instruments: Abingdon, UK). Multiple regions of interest within each area of ectopic mineral (10–16 regions per deposit) were analyzed, along with regions of interest in unaffected cortical bone within the same specimens (e.g., posterior aspect of the vertebrae). The elemental results were expressed as atomic percentages. Data were imported into GraphPad Prism (Version 6.01: San Diego, CA, USA) for statistical analysis. Data were assessed for normality using the Shapiro-Wilk test and for outliers using Grubbs’ two-sided test (*α* = 0.05: one result from cortical bone was identified). Data were then assessed using one-way ANOVA (*α* = 0.05) with Bonferroni’s multiple comparisons test.

The same tissues were assessed for X-ray diffraction patterns using a Bruker D8 Discover diffractometer (Bruker Co.: Billerica, MA, USA) and Bruker AXS general area detector diffraction system (Bruker AXS GmbH: Karlsruhe, DEU) at the Department of Earth Sciences, University of Western Ontario.^42^ The nominal beam diameter for each measurement was 300 µm. Regions of interest within the areas of ectopic mineral (5–7 regions per deposit) and unaffected cortical bone (1–2 regions) were selected for each motion segment using a microscope equipped with a charge-coupled device camera. Data were analyzed with Bruker’s DiffracPlus™ EVA software (Bruker Co.: Billerica, MA, USA) for comparison to powder diffraction files from the International Centre for Diffraction Data® (Newton Square, PA, USA) database as detailed in a previous protocol.^42^

## Supplementary information

Supplemental S2

Supplemental S1

## References

[1] Forestier J, Rotes-Querol J (1950). Senile ankylosing hyperostosis of the spine. Ann. Rheum. Dis..

[2] Resnick D, Niwayama G (1976). Radiographic and pathologic features of spinal involvement in diffuse idiopathic skeletal hyperostosis (DISH). Radiology.

[3] Resnick D, Shaul SR, Robins JM (1975). Diffuse idiopathic skeletal hyperostosis (DISH): forestier’s disease with extraspinal manifestations. Radiology.

[4] Mader R, Verlaan JJ, Buskila D (2013). Diffuse idiopathic skeletal hyperostosis: clinical features and pathogenic mechanisms. Nat. Rev. Rheumatol..

[5] Mata S (1997). A controlled study of diffuse idiopathic skeletal hyperostosis: clinical features and functional status. Med. (Baltim.).

[6] Utsinger PD (1985). Diffuse idiopathic skeletal hyperostosis. Clin. Rheum. Dis..

[7] Kuperus JS (2017). Classification criteria for diffuse idiopathic skeletal hyperostosis: a lack of consensus. Rheumatology.

[8] Weinfeld RM, Olson PN, Maki DD, Griffiths HJ (1997). The prevalence of diffuse idiopathic skeletal hyperostosis (DISH) in two large American Midwest metropolitan hospital populations. Skelet. Radiol..

[9] Westerveld LA (2008). The prevalence of diffuse idiopathic skeletal hyperostosis in an outpatient population in the Netherlands. J. Rheumatol..

[10] Julkunen H, Heinonen OP, Knekt P, Maatela J (1975). The epidemiology of hyperostosis of the spine together with its symptoms and related mortality in a general population. Scand. J. Rheumatol..

[11] Mader R, Novofestovski I, Adawi M, Lavi I (2009). Metabolic syndrome and cardiovascular risk in patients with diffuse idiopathic skeletal hyperostosis. Semin. Arthritis Rheum..

[12] Littlejohn GO (1985). Insulin and new bone formation in diffuse idiopathic skeletal hyperostosis. Clin. Rheumatol..

[13] Pillai S, Littlejohn G (2014). Metabolic factors in diffuse idiopathic skeletal hyperostosis—a review of clinical data. Open Rheumatol. J..

[14] Denko CW, Malemud CJ (2006). Body mass index and blood glucose: correlations with serum insulin, growth hormone, and insulin-like growth factor-1 levels in patients with diffuse idiopathic skeletal hyperostosis (DISH). Rheumatol. Int..

[15] Gorman C, Jawad ASM, Chikanza I (2005). A family with diffuse idiopathic skeletal hyperostosis. Ann. Rheum. Dis..

[16] Havelka S, Faberova R, Gatterova J, Trnavsky K (1990). Familial incidence of diffuse idiopathic skeletal hyperostosis. Vnitr. Lek..

[17] Kranenburg HC (2010). The dog as an animal model for DISH?. Eur. Spine J..

[18] Kranenburg H-JC, Hazewinkel HAW, Meij BP (2014). Naturally occurring spinal hyperostosis in dogs as a model for human spinal disorders. ILAR J..

[19] Warraich S (2013). Loss of equilibrative nucleoside transporter 1 in mice leads to progressive ectopic mineralization of spinal tissues resembling diffuse idiopathic skeletal hyperostosis in humans. J. Bone Miner. Res..

[20] Ii H (2016). Disruption of biomineralization pathways in spinal tissues of a mouse model of diffuse idiopathic skeletal hyperostosis. Bone.

[21] Resnick D (1978). Diffuse idiopathic skeletal hyperostosis (DISH) [ankylosing hyperostosis of Forestier and Rotes-Querol]. Semin. Arthritis Rheum..

[22] Verlaan JJ (2011). Quantitative analysis of the anterolateral ossification mass in diffuse idiopathic skeletal hyperostosis of the thoracic spine. Eur. Spine J..

[23] Mader R (2017). Diffuse idiopathic skeletal hyperostosis (DISH): where we are now and where to go next. RMD Open.

[24] Barsamian JG, Cobb LW, Bremer AM, Scheffer RB, Northup HM (1985). Radiographic, clinical, and histopathologic evaluation with surgical treatment of Forestier’s disease. Oral. Surg. Oral. Med. Oral. Pathol..

[25] Kuperus JS (2017). Histological characteristics of diffuse idiopathic skeletal hyperostosis. J. Orthop. Res..

[26] Kuperus, J. S. et al. The anterior longitudinal ligament in diffuse idiopathic skeletal hyperostosis: ossified or displaced? *J. Orthop. Res*. (2018). 10.1002/jor.24020. 10.1002/jor.24020 PMC6175084 29667228

[27] Fournier DE (2019). Ectopic spinal calcification associated with diffuse idiopathic skeletal hyperostosis (DISH): A quantitative micro-ct analysis. J. Orthop. Res..

[28] Chalmers J, Gray DH, Rush J (1975). Observations on the induction of bone in soft tissues. J. Bone Jt. Surg. Br..

[29] Boulman N, Slobodin G, Rozenbaum M, Rosner I (2005). Calcinosis in rheumatic diseases. Semin. Arthritis Rheum..

[30] Mader R, Verlaan JJ, Buskila D (2013). Diffuse idiopathic skeletal hyperostosis: clinical features and pathogenic mechanisms. Nat. Rev. Rheumatol..

[31] Oudkerk SF (2017). Diagnosis of diffuse idiopathic skeletal hyperostosis with chest computed tomography: inter-observer agreement. Eur. Radio..

[32] Slonimsky, E., Lidar, M., Stern, M. & Eshed, I. Degenerative changes of the thoracic spine do exist in patients with diffuse idiopathic skeletal hyperostosis: a detailed thoracic spine CT analysis. *Acta Radiol.* (2018). 10.1177/0284185118761205. 10.1177/0284185118761205 29482347

[33] Vernon-Roberts B, Pirie CJ, Trenwith V (1974). Pathology of the dorsal spine in ankylosing hyperostosis. Ann. Rheum. Dis..

[34] Fleish H, Neuman WF (1961). Mechanisms of calcification: role of collagen, polyphosphates, and phosphatase. Am. J. Physiol..

[35] Giachelli CM (1999). Ectopic calcification: gathering hard facts about soft tissue mineralization. Am. J. Pathol..

[36] Le Nihouannen D (2005). Ectopic bone formation by microporous calcium phosphate ceramic particles in sheep muscles. Bone.

[37] Ripamonti U (1996). Osteoinduction in porous hydroxyapatite implanted in heterotopic sites of different animal models. Biomaterials.

[38] Costa DO (2013). The differential regulation of osteoblast and osteoclast activity bysurface topography of hydroxyapatite coatings. Biomaterials.

[39] Kumaresan S, Yoganandan N, Pintar FA, Maiman DJ, Goel VK (2001). Contribution of disc degeneration to osteophyte formation in the cervical spine: a biomechanical investigation. J. Orthop. Res..

[40] Fornasier VL, Littlejohn G, Urowitz MB, Keystone EC, Smythe HA (1983). Spinal entheseal new bone formation: the early changes of spinal diffuse idiopathic skeletal hyperostosis. J. Rheumatol..

[41] Costa DO (2012). Control of surface topography in biomimetic calcium phosphate coatings. Langmuir.

[42] Flemming RL (2007). Micro X-ray diffraction (µXRD): a versatile technique for characterization of Earth and planetary materials. Can. J. Earth Sci..

