# Supplementary material for: Dystrophic calcification and heterotopic ossification in fibrocartilaginous tissues of the spine in diffuse idiopathic skeletal hyperostosis (DISH)
Source: Bone Res. 2020 Apr 2;8:16. doi: 10.1038/s41413-020-0091-6 (PMC7118090; doi:10.1038/s41413-020-0091-6)
Supplement: Supplementary file 1 — Supplemental S2 [file 41413_2020_91_MOESM1_ESM.docx]

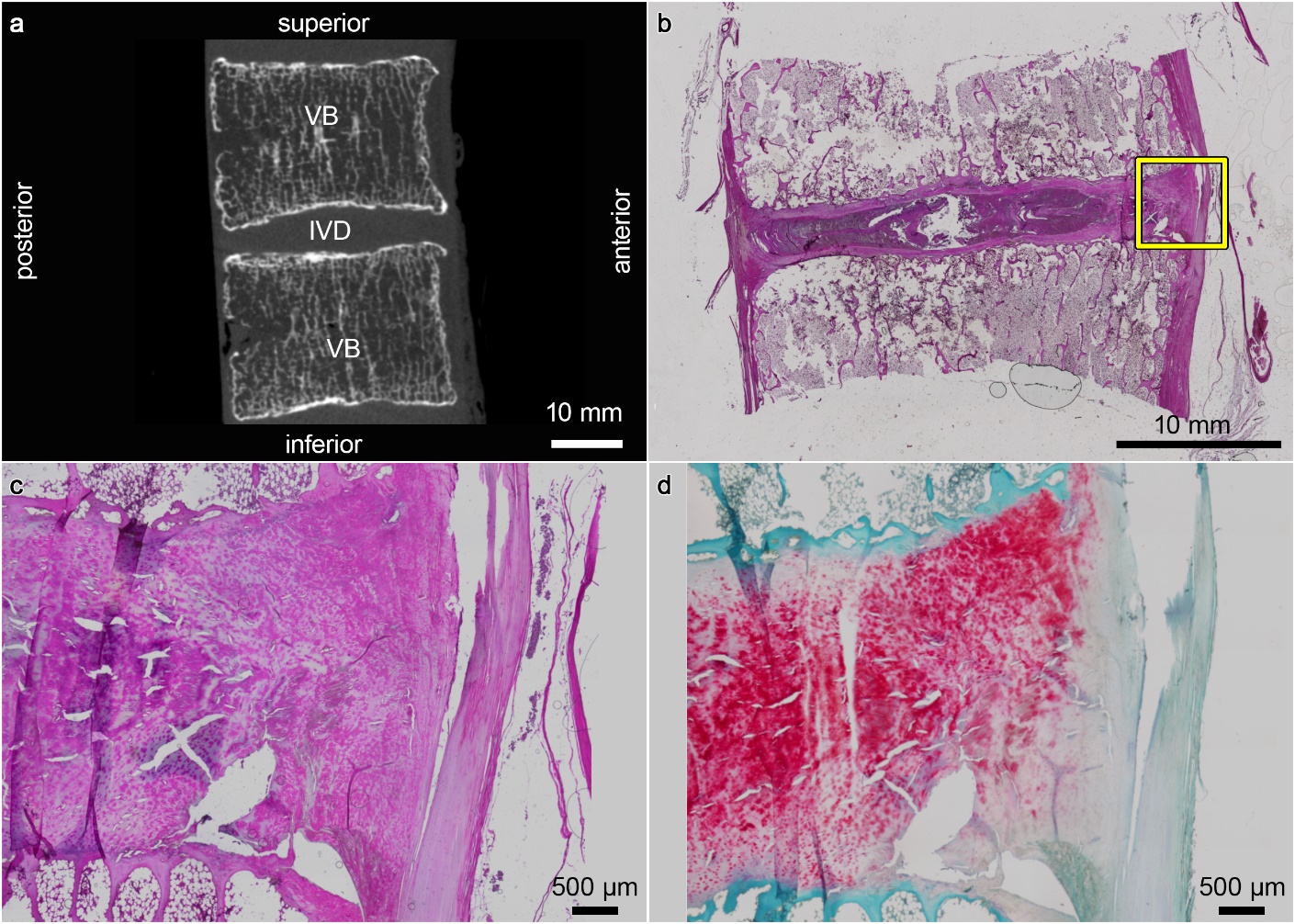


Supplement Figure S2. Histological features of age-matched spine without DISH.

Histological appearance of a representative motion segment (T8-9) from a 66 year-old-male donor without radiographic features associated with DISH. **a** Micro-computed tomographic sagittal section (voxel spacing 154 µm) of the intact motion segment showing the absence of ectopic mineral. VB, vertebral bone; IVD, intervertebral disc; scale bar represents 10 mm. **b** Representative mid-sagittal section stained with haematoxylin and eosin, scale bar represents 10 mm. Yellow box corresponds to the anterior region of the motion segment, shown below in **c** (stained with haematoxylin and eosin) and **d** (stained with Safranin O and Fast Green). All images are oriented as shown in **Panel a**.
