# Supplementary material for: Dystrophic calcification and heterotopic ossification in fibrocartilaginous tissues of the spine in diffuse idiopathic skeletal hyperostosis (DISH)
Source: Bone Res. 2020 Apr 2;8:16. doi: 10.1038/s41413-020-0091-6 (PMC7118090; doi:10.1038/s41413-020-0091-6)
Supplement: Supplementary file 2 — Supplemental S1 [file 41413_2020_91_MOESM2_ESM.docx]

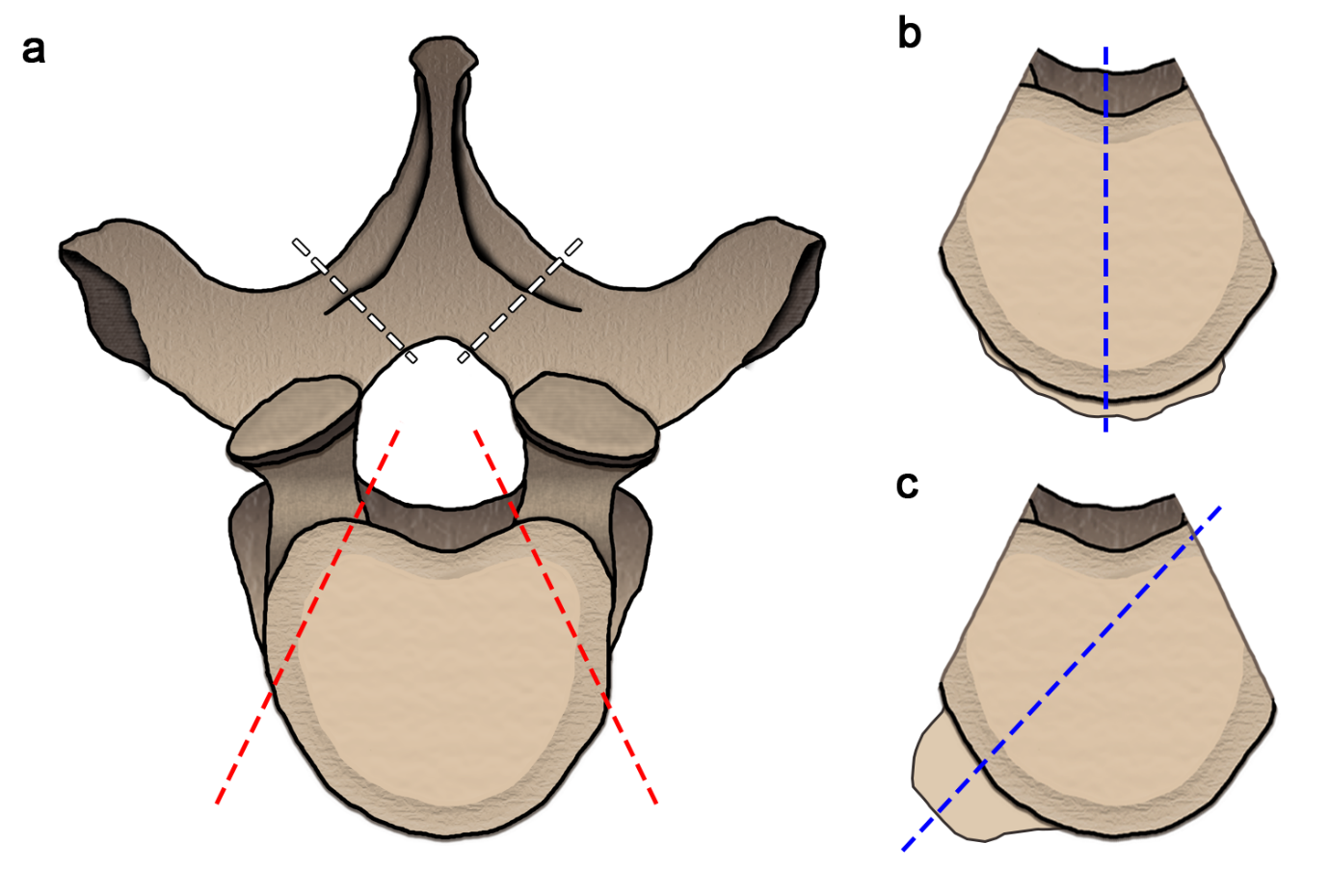


Supplement Figure S1. Schematic demonstrating the dissection approach.

Stylised images are superior views of a typical thoracic vertebrae. **a** Laminectomies corresponding to the white dotted lines were first performed to open up the posterior aspect of the vertebral foramen (i.e., spinal canal). Second, oblique cuts corresponding to the red dotted lines were performed medial to the costovertebral joints (as to preserve them) for the removal of the transverse processes. **b** The isolated vertebral body was then sliced sagittal through the center of the ectopic calcification/ossification, corresponding to the blue dotted line. **c** In cases of extreme right sidedness, oblique cuts through the center of the ectopic calcification/ossification was performed, corresponding to the blue dotted line. For subsequent analyses, a one mm slice was then carefully dissected from either one of the two halves before one was decalcified and the other stored.
